# Elementary Ophthalmic Optics

**Published:** 1902-12

**Authors:** 


**Elementary Ophthalmic Optics.** By J. HERBERT PARSONS, B.S., B.Sc. Pp. 162. London: J. & A. Churchill. 1901.

This book is intended to be used in conjunction with some other text-book on the refraction of the eye. Its subject is thus divorced to a certain extent from its practical application, and the usefulness of the work is likely to suffer in consequence. The first five chapters are devoted to the laws of reflection and refraction, and the information contained in them is hardly more easy to understand than that to be found in the ordinary text-books on optics. The chapter on ametropia is of much practical value, containing as it does explanations of the advantages of the proper position of spectacle lenses with relation to the cornea, and of the difference in the size of the retinal image in hypermetropia and myopia, with the consequent difficulty in prescribing lenses for anisometropia. The chapters on the ophthalmoscope and retinoscopy show evidence of great care in arrangement and minute attention to details. As an example of this we would refer to the explanation of the shadow margin in retinoscopy. The table of contents is very brief, and there is no index. For purposes of reference this is a distinct disadvantage; but the omission is to a certain extent compensated for by printing all the more important propositions in italics.

